# Supplementary material for: Characterization of Hyaluronidase 4 Involved in the Catabolism of Chondroitin Sulfate
Source: Molecules. 2022 Sep 18;27(18):6103. doi: 10.3390/molecules27186103 (PMC9501593; doi:10.3390/molecules27186103)
Supplement: Supplementary file 1 [file molecules-27-06103-s001.zip › molecules-1753585-supplementary.pdf]

molecules

MDPI

Supplementary Materials

Characterization of Hyaluronidase 4 Involved in the Catabolism of Chondroitin Sulfate

Shuhei Yamada * and Shuji Mizumoto

Department of Pathobiochemistry, Faculty of Pharmacy, Meijo University, 150 Yagotoyama, Tempaku-ku, Nagoya 468-8503, Japan
* Correspondence: shuheiy@meijo-u.ac.jp; Tel.: +81-52-839-2650

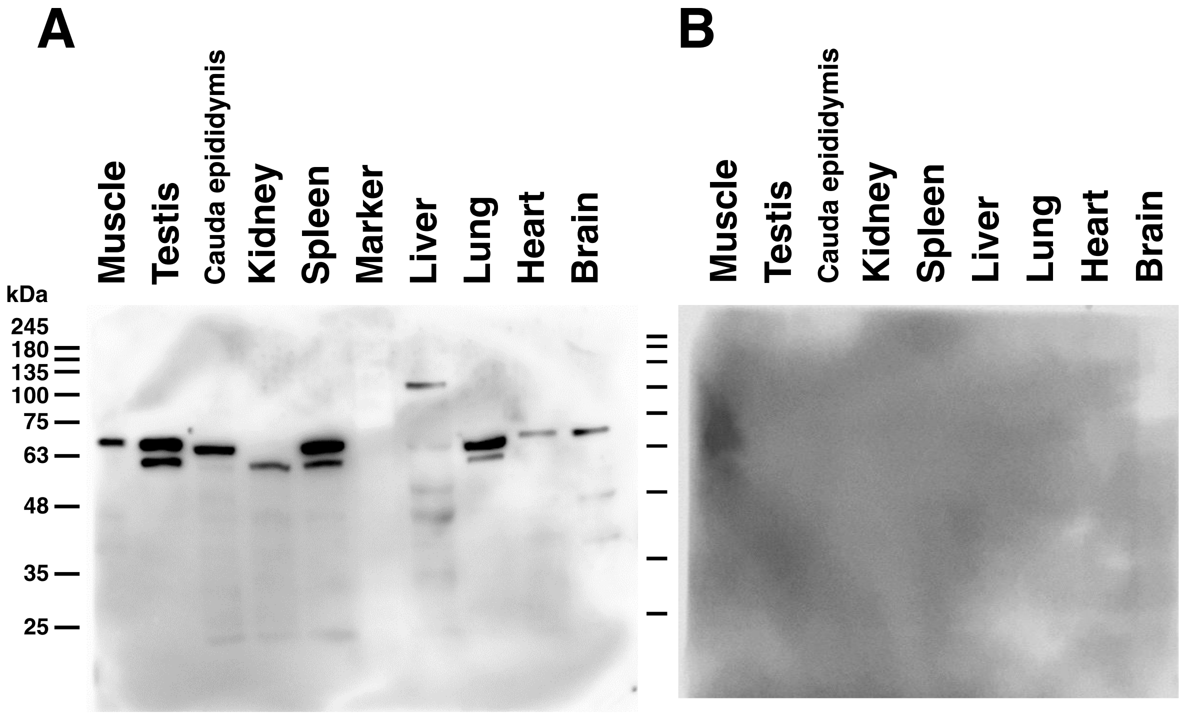

Supplementary Figure S1. Tissue distribution analysis of rat HYAL4 protein in various rat tissues. Expression levels of rat HYAL4 protein in various tissues of a Wistar rat were examined by Western blotting using an anti-HYAL4 antibody (A-7), HRP-conjugate (A). HRP-conjugated normal mouse IgG was used instead of the primary antibody for the control experiments (B).

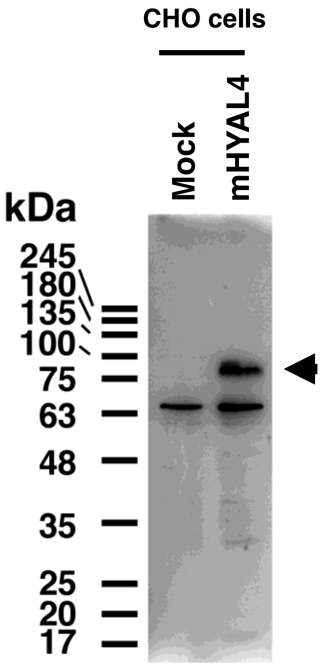

Supplementary Figure S2. Western blotting of whole cell lysate of CHO cells stably transfected with or without the *mHyal4* gene is shown using an anti-HYAL4 antibody (A-7) and anti-mouse IgG conjugated with HRP. Higher expression of HYAL4 protein at around 70 kDa was detected in the overexpressing cells. In addition, a band indicated by an arrowhead was found, considered to be the HYAL4 protein, which might be highly modified post-translationally. When CHO cells stably transfected with *mHyal4* were subjected to cellular localization analysis, both bands were detected in the organelle and membrane proteins fraction (results not shown).
